# Can a Low-Phosphate Diet for Chronic Kidney Disease Treat Cancer? An Interdisciplinary Literature Review

**DOI:** 10.3390/medicines11020005

**Published:** 2024-01-30

**Authors:** Ronald B. Brown, Philip Bigelow

**Affiliations:** School of Public Health Sciences, University of Waterloo, Waterloo, ON N2L 3G1, Canada; philip.bigelow@uwaterloo.ca

**Keywords:** cancer, low-phosphate diet, phosphate toxicity, chronic kidney disease, ketogenic diet, fasting

## Abstract

**Background:** Cancer therapeutics have a low success rate in clinical trials. An interdisciplinary approach is needed to translate basic, clinical, and remote fields of research knowledge into novel cancer treatments. Recent research has identified high dietary phosphate intake as a risk factor associated with cancer incidence. A model of tumor dynamics predicted that reducing phosphate levels sequestered in the tumor microenvironment could substantially reduce tumor size. Coincidently, a low-phosphate diet is already in use to help patients with chronic kidney disease manage high serum phosphate levels. **Methods:** A grounded-theory literature-review method was used to synthesize interdisciplinary findings from the basic and clinical sciences, including oncology, nephrology, nutritional epidemiology, and dietetic research on cancer. **Results:** Findings of tumor remission associated with fasting and a ketogenic diet, which lower intake of dietary phosphate, support the hypothesis that a low-phosphate diet will reduce levels of phosphate sequestered in the tumor microenvironment and reduce tumor size. Additionally, long-term effects of a low-phosphate diet may reverse dysregulated phosphate metabolism associated with tumorigenesis and prevent cancer recurrence. **Conclusions:** Evidence in this article provides the rationale to test a low-phosphate diet as a dietary intervention to reduce tumor size and lower risk of cancer recurrence.

## 1. Introduction

More than half a century after the U.S. National Cancer Act of 1971 declared war on cancer, Castuera wrote in the American Journal of Economics and Sociology that the war on cancer failed because its main focus was on increasing technological advancements in cancer research rather than developing more programs to save lives [1]. The decades following 1971 saw a continued rise in cancer deaths until a reversal of cancer mortality during the mid-1990s which was “based more on inexpensive anti-smoking and other prevention programs than on expensive treatment methods” [1]. By 2014, the International Agency for Research on Cancer (IARC) declared that the battle against cancer will not be won with treatments alone and that measures to effectively prevent a global crisis in cancer are needed [2].

However, the majority of international cancer research funding continues to be directed toward cancer treatments, while prevention remains the smallest investment category [3]. Yet, current treatments have significant clinical limitations. For example, a recent review of cancer chemotherapies over the past six decades found that the rate of outcome failures for solid tumors ranged between 85 and 95% [4]. Drug therapies cause more harm than benefits for some patients [5], and cancer drugs licensed by the U.S. Food and Drug Administration (FDA) and the European Medicines Agency (EMA) from 2003 to 2013 improved overall patient survival by an average of only 3.43 months compared to treatments in 2003 [6].

Prescribing higher doses of chemotherapy to cancer patients often provides few improved outcomes while increasing toxicity and harm to patients’ quality of life [7], and large amounts of funding are invested in cancer treatment studies with questionable pretrial data [8]. Consequently, the latest drugs and technologies to treat cancer globally will not increase survival, and greater advocacy is needed for less expensive and more effective cancer treatments, “especially in low- and middle-income countries” [9]. Due to changes in lifestyle and increases in the ageing population, cancer is estimated to affect 21.7 million people by 2030 at a cost of USD 458 billion [9]. Effects of financial toxicity experienced by more than half of U.S. patients treated for cancer include “house repossession, bankruptcy, loss of independence, and relationship breakdowns” [10]. Additionally, “Cancer therapeutics currently have the lowest clinical trial success rate of all major diseases”, and “cancer will soon be the leading cause of mortality in developed countries” [11].

But the situation is not hopeless. Researchers can target the development of simple, inexpensive, effective, accessible, and equitable treatments and prevention strategies to address the increasing social and economic burden of global cancer [9]. “Lateral thinking, rather than an approach directed to a particular cancer, has produced the most breakthroughs historically” [1], and “discoveries in medicine have often come from the most remote and unexpected fields of science” [12]. An abundance of new approaches to cancer can be provided by a collaboration of scientists conducting cross-disciplinary research [13]. According to the National Academies of Sciences, Engineering, and Medicine, interdisciplinary research methods can “solve problems whose solutions are beyond the scope of a single discipline or field of research practice” [14].

The purpose of the present interdisciplinary literature review is to synthesize findings from clinical and basic science research in oncology, cancer biology, biochemistry, nephrology, nutritional epidemiology, and dietetics. The results propose a novel treatment for cancer using a low-phosphate diet already in use to manage dysregulated serum phosphate in patients with chronic kidney disease (CKD). The essential mineral phosphorus is acquired mainly through the diet in the form of phosphate (chemical formula PO_4_) and concentrations of inorganic phosphate (Pi) are regulated in the blood serum by a network of endocrine hormones involving the kidneys, bone, parathyroid glands, and intestines [15]. Dysregulation of phosphate metabolism causes phosphate toxicity in the tissues of the body [16].

This narrative review explores the interdisciplinary relationship of cancer with renal disease and proposes that dysregulated metabolism of Pi is a common mediating factor in the etiology of both diseases. A grounded theory literature-review method was used to purposely search, select, analyze, and compare research findings from online databases like Pub Med and Google Scholar to synthesize new knowledge, insights, and new directions for further research [17]. Findings reviewed in this paper provide the rationale to hypothesize that a low-phosphate dietary intervention administered to patients with CKD will be efficacious in the remission of solid tumors and prevention of tumor recurrence in patients with cancer.

## 2. Diet and Cancer

In an “Introduction to Dietary Research and Cancer” [18], Gacche wrote “Epidemiological studies have clearly demonstrated the fact that diet and nutrition have profound impact and influence on the progression of cancer and also on risk of developing cancers”.

Yet, the author also points out gaps in the clinical application of this information such as the need for more evidence from rigorous clinical research and the low impact of nutritional advice due to “moderate interest of clinical oncologists in diet and nutritional interventions”. Of importance, patients who confront cancer become much more aware of the value of their health and are highly motivated to do “everything in their power to increase their chance of survival” [19]. Cancer patients also have a high interest in dietary strategies, and a review of popular diets found that almost half of surveyed cancer patients were sufficiently motivated to change their dietary habits “with the hope that they will improve survival and prevent recurrence” [20]. Information in the present review should be of interest to a broad audience of cancer patients, oncologists, and nutritionists.

Among topics in emerging research on diet and cancer, Gacche described “the diet/metabolite-mediated regulation of cancer signaling pathways/growth factors” as well as metastasis and immunosurveillance in cancer biology. Importantly, metabolomics, the study of chemical metabolites derived from metabolic processes “offers an opportunity to develop a biomarker-based approach to dietary assessment in cancer epidemiology” [21]. A 2023 review of nutritional metabolomics in the association of diet and breast cancer described a wide variety of metabolites from fat, protein, carbohydrate, and other nutrients, but significantly, no mention was made of metabolites related to dietary phosphate [22]. Yet, circulating serum Pi derived from dietary phosphorus absorbed in the intestines “functions as a constituent of cellular metabolites” [23].

Accumulation of interstitial Pi in the tumor microenvironment has been identified as a biomarker of cancer progression (metastasis) [24]. Recently, Fu et al. used animal models to lower Pi concentrations in tumors by administering the phosphate binder lanthanum acetate, and the researchers suggested that this procedure might provide a new anticancer strategy to reduce tumor growth and metastatic progression [25]. Lv et al. used the phosphate binder sevelamer in a rabbit model to lower Pi concentrations in tumor grafts, which improved transarterial chemoembolization that blocks the tumor blood supply while increasing tumor accumulation of the chemotherapeutic agent doxorubicin [26]. Bi et al. [27] lowered Pi-induced metabolic stress by using transarterial sevelamer embolization to “occlude the tumor-feeding vessel” and deplete tumor Pi concentrations, leading to inhibition of liver cancer progression in a rabbit model. Furthermore, animal studies have shown that high dietary phosphate increases lung tumorigenesis through cell signaling in the phosphoinositide 3-kinase (PI3K)/Akt/mTOR signaling pathway [28]. A high-phosphate diet fed to mice also increased skin tumorigenesis through the N-ras-extracellular signal-regulating kinase 1/2 (ERK1/2) pathway that regulates cell proliferation [29].

Additionally, obesity is a risk factor for cancers in at least 13 sites in humans, and obesity has been associated with increased dietary phosphate and elevated serum levels of hormones that regulate Pi metabolism: fibroblast growth factor 23 (FGF23) and parathyroid hormone (PTH) [30]. Obesity is also associated with increased intake of ultra-processed foods that are high in phosphate additives, including phosphoric acid in colas [30]. A 2022 systematic review and meta-analysis found that obesity was associated with an increased risk of mortality in prostate cancer [31], and an earlier analysis of 47,885 men in the Health Professionals Follow-Up study found increased risk of lethal and high-grade prostate cancer associated with dietary phosphorus intake [32]. Another 2022 meta-analysis involving 669,080 participants in case–control studies found that high intake of dietary phosphorus and high serum phosphorus concentrations were associated with an 8% and 7% increase in prostate cancer risk, respectively [33]. Higher levels of serum prostate specific antigen (PSA), a biomarker for prostate cancer, were also associated with an intake of dietary phosphorus above 1151 mg per day in a secondary analysis of data from the U.S. National Health and Nutrition Examination Survey (NHANES), 2003–2010 [34].

In 2023, the present authors published a review proposing that the association of alcohol consumption with increased risk of breast cancer is mediated by hyperphosphatemia caused by alcohol-induced rhabdomyolysis which releases intracellular phosphate from skeletal muscle into the serum [35]. Also in 2023, the authors found that a 2.30 relative risk for breast cancer incidence in a cohort of middle-aged U.S. women was associated with >1800 mg dietary phosphorus compared to 800–1000 mg phosphorous recommended by the U.S. National Kidney Foundation for patients with CKD (RR: 2.30; 95% CI: 0.94–5.61; *p* = 0.07) [36]. Although the study’s small cohort likely reduced statistical significance, these results are supported by evidence in the present review, and further clinical investigations are warranted to test the hypothesis that limiting cancer patients to 800–1000 mg or less of phosphorus a day will reduce and prevent cancer promotion and progression. Furthermore, another 2023 study of the cohort by the present authors found that a greater magnitude of abnormal bone mineral density changes (mineral deposition in osteosclerosis followed by mineral loss in osteoporosis) was associated with women self-reporting breast cancer incidence compared to women remaining cancer-free [37]. This finding implicates phosphate toxicity as a potential contributing factor to bone metastases in metastatic breast cancer, which should also be investigated with a low-phosphate dietary intervention.

In contrast to the above findings, a recent analysis of data from the Prostate, Lung, Colorectal and Ovarian Cancer Screening Trial found that dietary phosphorus was not significantly associated with risk of pancreatic cancer in adults [38]. The study examined associations of pancreatic cancer with daily recommended dietary allowances for calcium, magnesium, and phosphorus (700 mg phosphorus [39]) compared to deficient intake of these nutrients. Importantly, effects of excessive nutrient intake were not investigated, and the researchers added “It is biologically plausible that phosphorus is implicated in pancreatic carcinogenesis”. Furthermore, calcium intake in the study was only associated with reduced cancer risk when fat was also consumed, which may be due to the consumption of full-fat dairy products that are high in calcium but lower in phosphorus per calorie compared to low-fat or nonfat dairy products.

## 3. Pi and Molecular Mechanisms in Tumorigenesis

Hallmarks of cancer metabolism include increased demand for nutrients to support rapid tumor growth and cell proliferation [40,41]. However, evidence of Pi molecular mechanisms in tumorigenesis suggests an opposite dynamic relationship between supply and demand for growth-promoting nutrients. That is, rather than demanding more nutrients for growth, tumorigenesis is associated with a dysregulated oversupply of growth-promoting dietary phosphorus.

Phosphate bonded to deoxyribose forms the backbone of nucleic acids, deoxyribonucleic acid (DNA) and ribonucleic acid (RNA), which direct the genetic expression of proteins in cell proliferation [42]. A genetic code transcribed from the cell nucleus is transported via messenger RNA (mRNA) to the cell ribosomes for biosynthesis of cell proteins. Early animal research shows that a high-phosphorus diet induced hyperphosphatemia, increased biosynthesis of mRNA, and stimulated hyperplasia in the parathyroid glands, one of the organs that regulates phosphorus metabolism [43]. Researchers also described how liver tumorigenesis was delayed in precancerous tissue when phosphorus incorporation into cellular nucleic acids was depressed [44]. Phosphorus in RNA also contributes significantly to a higher total biomass of phosphorus in malignant tissue compared to normal tissue [45].

Exposure to high Pi levels increases expression of genes in cancer cells that promote angiogenesis and neovascularization which supply blood to the tumor [46]. Additionally, sodium phosphate cotransporter 2b (NaPi2b) is highly expressed in cancer cells of the ovary, lung, thyroid, and breast compared to normal tissue [47]. By comparison, H^+^-dependent Pi transport in breast cancer cells is five times higher than Na^+^-dependent Pi transport when the cells are exposed to high extracellular levels of Pi [48], facilitating sequestration of excessive phosphate into the tumor.

A biologically plausible advantage of Pi sequestration in tumorigenesis is that it removes potentially lethal and tissue-damaging amounts of serum Pi circulating throughout the body, which may explain why immune system responses appear to protect tumors in putative “tumor immune evasion” [49]. For example, a model with mice that overexpress the tumor-suppression protein P53 reduced tumorigenesis but increased cachexia, including sarcopenia, organ atrophy, skeletal kyphosis, and premature death. These cachexic effects are similar to effects from phosphate toxicity in a model of mice lacking klotho, a regulator of phosphate metabolism [50]. Consequently, if a tumor is destroyed with conventional treatments like radiation or chemotherapy, rapid release of large amounts of intracellular Pi and other cellular constituents into the serum can cause an oncologic emergency known as tumor lysis syndrome [51]. Moreover, surgical removal of a primary tumor is associated with increased tumor recurrence and metastasis [52], which implies a plausible protective response that persistently sequesters dysregulated Pi into tumors.

Tumor progression is also associated with inflammation [53], and inflammation in hemodialysis patients is strongly correlated with serum phosphorus levels [54], suggesting that dysregulated phosphate metabolism and hyperphosphatemia are potential mediating factors in the association of inflammation with tumor progression [55]. Additionally, hyperphosphatemia in hemodialysis patients with end-stage renal disease (ESRD) is associated with increased proliferation and complexity of bacterial flora in the gut microbiota compared to controls without ESRD, but increased proliferation ceased as intestinal phosphorus levels were lowered with phosphate binders [56]. Furthermore, “dysbiosis of gut bacteria, fungi, viruses and Archaea accompanies colorectal tumorigenesis” [57]. The association of intestinal cancer risk with flora dysbiosis may be mediated by high phosphate levels that stimulate microbial overgrowth, and research should investigate the use of phosphate binders or a low-phosphate diet to reduce intestinal phosphate levels, lower the risk of tumorigenesis, and restore normal balance to the gut microbiota.

## 4. Dietary Recommendations to Reduce Cancer Risk

Describing the general lack of policies for cancer prevention based on nutrition and lifestyle, Kerschbaum and Nüssler pointed out that 30–50% of cancers can be prevented through diet and lifestyle modifications [58], and the researchers cited findings of the World Cancer Research Fund (WCRF). Recommendations of the WCRF/American Institute for Cancer Research (AICR) prioritize future research on nutrients and the metabolism of the tumor microenvironment [59]. This future research direction has special relevance for research of phosphate toxicity and cancer. For example, Table 1 lists WCRF/AICR recommendations to prevent cancer, with additional associations based on cited research findings for phosphate toxicity.

An analysis of cohort data from the Iowa Women’s Health Study reported that “better adherence to the WCFR/AICR cancer prevention guidelines is associated with a lower risk of postmenopausal breast cancer” [67]. Yet, the researchers did not find an association between the specific diet recommendations and incidence of breast cancer, perhaps due to the dietary recommendation allowing unrestricted amounts of high-phosphate foods like wholegrains, legumes, nuts, seeds, and tubers. Nevertheless, other studies found reduced risk of breast cancer with greater adherence to the overall WCFR/AICR cancer prevention guidelines, including Hastert et al. [68]; Harris, Bergkvist, and Wolk [69]; Makarem et al. [70]; and Catsburg, Miller, and Rohan [71].

Related to dietary patterns in cancer, Gacche [18] wrote that the epidemiological evidence is unconvincing that a particular dietary pattern reduces cancer “prognosis, recurrence and mortality”. The author notes however that overall, for a variety of cancers, “the Western dietary pattern seems to be detrimental”. Gacche also expressed surprise that many of the world’s top hospitals and healthcare agencies are unable to provide consistent answers to questions asked by cancer patients regarding “the optimal diet during and after cancer treatments,” and the author suggested that the scientific community “undertake research activity for resolving the questions being asked by cancer patients”. With over 1000 registered clinical trials investigating the effect of dietary factors on cancer, no dietary interventions have received FDA approval for prevention of cancer, and Gacche suggested focusing research on “molecular mechanisms” and “impact on current chemotherapy efficacy” through dietary adjuvant therapies [18].

## 5. Diet Therapy for Hyperphosphatemia

Dysregulated serum phosphate in hypophosphatemia (low serum Pi) is common in cancer patients, but “it is important to be aware of pseudohypophosphatemia, defined as spuriously low serum phosphorus values that do not correspond to their actual systemic levels” [72]. Osuka and Razzaque noted that “most of the body phosphate is present in the bone as hydroxyapatite, and only 1% of the total phosphate is available in the extracellular compartment” [73]. The authors added that “serum measurements of extracellular phosphate therefore reveal only a tiny fraction of total body phosphate and might not always reflect the amount of phosphate uptake and its distribution”. For example, hypophosphatemia in cancer patients can be caused by a shift of serum phosphate into the intracellular compartment of tumors during tumor genesis syndrome [74].

Dysregulated serum phosphate is also common in renal disease. In 2021, an analysis of mineral metabolism markers in hemodialysis patients from Taiwan with ESRD found that a short-term increase in dietary phosphorus intake of only 100 mg was associated with a statistically significant serum phosphate increase of 0.28 mg/dL [75]. In a 2023 study of Taiwan hemodialysis patients with ESRD, Tsai et al. found that serum phosphate levels rapidly declined within 2 days of lowering dietary phosphate by 300 mg to an average of 520 mg phosphate (SD 104 mg) [76].

Additionally, high pill burden in hemodialysis patients (about half of the pills from phosphate binders) averages 19 pills per day which is associated with “poor adherence, higher serum phosphate levels, and impaired health-related quality of life,” implying a need to further emphasize control of dietary phosphate intake [77]. Education and planning talks were provided to hemodialysis patients who identified barriers to long-term management of hyperphosphatemia, including the need to tailor phosphate binder prescriptions to their eating habits and the need for sufficient resources on diet change, and the talks were successful in reducing patients’ mean serum phosphate levels by 0.31 mg/dL [78].

Education in hyperphosphatemia management for hemodialysis patients is enhanced by dietitian-led guidance in renal diet education [79]. In addition to clinical improvements, educational programs in management of hyperphosphatemia have potential for significant savings in healthcare costs [80]. A 2020 systematic review and meta-analysis of clinical trials found that 20–30 min of phosphate-specific diet therapy provided monthly by a dietitian to patients on hemodialysis with persistent hyperphosphatemia significantly lowered serum phosphate for 4–6 months without negatively affecting nutritional status [81]. Another systematic review in 2021 examined patient adherence to phosphate control through educational or behavioral interventions and found that outcomes included improved serum phosphate levels in patients and better knowledge, self-efficacy, and adherence to phosphate control methods [82].

Because dietary sources of phosphate have different levels of bioavailability, researchers suggested that indexing foods by degree of phosphate bioavailability (Phosphatemic Index) could assist patients with CKD in food selection for phosphate management [83]. However, an important limitation of this approach is that phosphate in the form of phytates in plant-based foods like legumes and grains can bind with calcium in the intestines and reduce calcium absorption, potentially contributing to disturbances in calcium homeostasis [84].

### Vitamin D and Hyperphosphatemia

The bioactive form of vitamin D, 1,25-dihydroxyvitamin D3 [1,25(OH)_2_D] or calcitriol, is biosynthesized by the kidneys from the storage form of vitamin D, 25(OH)D, and calcitriol increases dietary phosphate absorption in the intestines to increase serum Pi levels [85]. If serum Pi levels are high, the kidneys produce less calcitriol. This regulatory mechanism potentially explains why bioactive vitamin D levels drop in patients with CKD [86] and in patients with cancer [87], potentially related to rising serum Pi levels in hyperphosphatemia. Additionally, the inability to reduce cancer risk with vitamin D supplements in patients with cancer may be explained by failure to address the underlying cause of dysregulated Pi in tumorigenesis [65]. Moreover, calcitriol is associated with “antiproliferative, pro-apoptotic, anti-cell migration and antiangiogenic activity in a number of preclinical studies in many different cancer types” [87], but these associations could be mediated by reduced serum phosphate levels. For example, a low-phosphate diet was found to restore normal calcitriol levels in patients with CKD [88], and future research is needed to investigate how a low-phosphate diet with reductions in serum phosphate may increase calcitriol levels in patients with cancer.

## 6. Cancer Cachexia, Protein, and Dietary Phosphate

Cachexia in cancer patients from sarcopenia or loss of skeletal muscle mass is more complicated than simply being a problem of insufficient nutrient intake, and “nutrition alone cannot reverse cachexia” [89]. A negative balance of protein and energy in cachexia is “driven by a variable combination of reduced food intake and abnormal metabolism” [90], suggesting a role for dysregulated phosphate metabolism. For example, in 2018, researchers from the Republic of China found that serum phosphate levels within the high end of the normal range were associated with age-related loss of muscle strength, or dynapenia, in an analysis of U.S. NHANES data [91]. Additionally, a 2023 study in the Journal of Cachexia, Sarcopenia and Muscle found that feeding geriatric mice a low-phosphate diet lowered the serum phosphate of the mice to levels approaching serum levels in younger mice [92]. Remarkably, compared to geriatric mice fed a standard diet three-times higher in phosphate, the muscle mass measured in the gastrocnemius and tibialis of the geriatric mice receiving the low-phosphate diet increased by 44%, along with increases in strength and physical performance. These findings help ameliorate the concern that a low-phosphate diet for cancer patients with cachexia might increase sarcopenia.

Nevertheless, dietary recommendations and specific nutrients needed to avoid muscle loss in patients with sarcopenia and cancer cachexia are rarely based on evidence from clinical trials [89]. For example, the European Society for Clinical Nutrition and Metabolism (ESPEN) recommends a daily dietary protein intake for cancer patients of between 1.0 and 1.5 g/kg body weight [93], but “evidence-based studies to support the optimal quantity are largely missing” [94]. Furthermore, a gram of protein on a mixed diet contains an average of 12–14 mg phosphorus [95]. Therefore, at a body weight of 154 pounds (~70 kg), the ESPEN protein recommendations range from 70 g to 105 g which calculates to between 840 mg and 1470 mg of phosphorus, approximating the level of >1400 mg phosphorus intake associated with increased all-cause mortality in a 2013 analysis of data from the U.S. NHANES III [96]. Alternatively, a 2020 review suggests that a plant-based low-protein diet, with 0.6–0.8 g protein per kg body weight, is more conservative, effective, and safe when “administered by dietitians trained in non-dialysis CKD care” [97]. This lower level of recommended protein at 70 kg body weight ranges between 42 g and 56 g of daily protein intake, which calculates to between 504 mg and 784 mg of dietary phosphorus. The 2020 updated guidelines from the U.S. National Kidney Foundation also confirms that a low-protein diet (0.55–0.60 g/kg body weight) in combination with sufficient energy intake is safe for nondialysis adults and patients with CKD [98].

## 7. Low-Phosphate Diet for Cancer Treatment

Phosphoproteins in animal-based foods (casein in bovine milk and ovo-vitellin in egg yolk [99]) are proteins with attached phosphate groups [100], and phosphorylated proteins are associated with cancer and metabolic disorders [101]. Conversely, lower amounts of phosphoproteins in plant-based diets may contribute to a reduced cancer risk associated with plant-based dietary patterns [102]. Additionally, phosphate stored in plants as phytates have lower bioavailability compared to phosphate in animal-based foods [103].

Unlike dietary phosphoproteins, dietary lipids lack phosphorus, which explains why ketogenic diets that are high in lipids tend to be lower in overall dietary phosphorus levels. For example, in 2022, Hagihara et al. administered a high-lipid/low-carbohydrate ketogenic diet to patients with advanced cancer and used positron emission tomography–computed tomography (PET-CT) to document remission of tumors that occurred in patients [104]. Based on examples of daily menus provided in the study’s supplementary material, the daily phosphorus content of the ketogenic formula administered to the patients (2.4 mg phosphorus per gram ketogenic formula) totaled 582.7 mg phosphorus. This amount of phosphorus is similar to the average of 520 mg previously cited in the 2023 Taiwan study by Tsai et al. which rapidly reduced serum phosphate levels in hemodialysis patients with ESRD [76]. These dietary phosphorus levels are also lower than the dietary reference intake (DRI) of 700 mg for adults and approximately equal the estimated average requirement (EAR) of 580 mg for adults [39]. Moreover, the ketogenic formula provides an amount of dietary phosphorus that is much lower than the average daily intake of 1189 mg for U.S. adult women and 1596 mg for U.S. adult men [64].

Although animal-based proteins in the ketogenic formula used by Hagihara et al. are generally high in phosphorus, the overall low phosphorus content of the diet is likely due to high lipid intake and restricted intake of carbohydrate foods that contain high levels of phosphorus like starchy vegetables, legumes, tubers, and grains. Hagihara et al. also reported that “total cholesterol and low-density lipoprotein cholesterol levels were significantly increased” in cancer patients [104], likely related to the high intake of dietary cholesterol in the diet.

Of concern, low-carbohydrate diets with animal-based protein and fat are generally associated with higher mortality [105]. Long-term compliance with the ketogenic diet is also challenging for patients, and the diet can increase risks of cardiovascular disease [106]. Interestingly, while a dietary pattern with a relatively higher intake of cholesterol is associated with increased cardiovascular disease risk, the relatively lower phosphate intake in this dietary pattern reduces cancer risk, which likely explains the inverse relationship between atherosclerosis and cancer in the medical literature [107]. The ketogenic diet may also induce inflammation and oxidative stress and accelerate aging, and adverse effects of the diet include muscle pain, flu-like symptoms, low energy, hepatic insulin resistance, and micronutrient deficiencies [108]. Furthermore, a ketogenic diet tested in a mouse model of cancer cachexia delayed tumorigenesis but also reduced the cancer survival time and caused more rapid development of cachexia related to reduced synthesis of corticosterone [109]. For comparison purposes, a similar mouse model of cachexia could test effects of a diet containing normal amounts of macronutrients (carbohydrates, protein, and fat) with reduced amounts of phosphate.

The rationale for the hypothesis that the low-carbohydrate component of the ketogenic diet will reduce tumor size is based on the Warburg effect, which states that tumors are dependent on anaerobic metabolism of glucose [110]. But drastically restricting carbohydrate dietary intake has little effect on serum levels of glucose due to the compensatory release of glucose from glycogenolysis and de novo synthesis of glucose from the breakdown of lactate, glycerol, and glucogenic amino acids in gluconeogenesis, even during fasting [111]. Moreover, consumption of carbohydrates by a very large tumor weighing half a kilogram “would account for only 9.8 g/day, a negligible percentage of the total glucose disposal of the body” [112]. The evidence suggests that therapeutic effects of the ketogenic formula administered by Hagihara et al. to patients with advanced cancer are due more to lower dietary phosphorus levels rather than to lower dietary glucose levels.

Overall, the ketogenic diet is an inefficient, over-restrictive, unhealthy, nutritionally imbalanced, and unsafe diet to temporarily lower dietary phosphate intake for cancer treatment. “For most individuals, the risks of such diets outweigh the benefits” [106]. No major cancer agency has recommended ketogenic diets for either prevention or treatment of cancer [113]. Alternatively, a low-phosphate diet with a proper balance of mostly plant-based proteins, healthy plant-based lipids, unrefined carbohydrates, and other nutrients associated with health benefits offers patients more food choices with possibly more satisfaction and better compliance and may have higher therapeutic prospects for long-term success in reducing cancer risk [102].

### 7.1. Fasting for Cancer Treatment

“Emerging evidence suggests that fasting could play a key role in cancer treatment by fostering conditions that limit cancer cells’ adaptability, survival, and growth. Fasting could increase the effectiveness of cancer treatments and limit adverse events. Yet, we lack an integrated mechanistic model for how these two complicated systems interact, limiting our ability to understand, prevent, and treat cancer using fasting” [114]. Intermittent fasting, in which food intake is restricted to limited feeding periods, is another dietary approach that has potential to improve cancer treatment efficacy and quality of life for patients, but clinical trials have not vetted the effects of intermittent fasting for clinical use [115]. Water-only fasting was associated with remission of follicular lymphoma in a case study of a 42-year-old woman, and the malignancy did not reoccur during a three-year follow-up period during which the woman strictly followed a whole-food plant-based diet [116]. The researchers suggested that “fasting affects cancer cells by reducing nutrients necessary for sustained growth”.

Importantly, when fasting or a fasting-mimicking diet was combined with chemotherapy to reduce tumor size, “the necrotic/apoptotic areas were not expanded compared with chemotherapy alone and tumor tissue showed decreased proliferation markers, meaning that the dietary intervention acted by preventing cells from proliferating rather than killing them” [117]. This finding supports an earlier hypothesis by Schipper, Turley, and Baum proposing that cancer is caused by dysregulated metabolism which may be reversible, and the researchers suggested that “killing strategies may be counter productive” in treating cancer [118]. “Ideally, one would like a treatment that eradicated the tumor, not necessarily by killing but by reregulation which may lead to restoration of normal patterns of cell death (apoptosis) or reversion to a resting proliferative state” [118].

As in the ketogenic diet, one of the effects of fasting for treatment of cancer is attributed to dietary glucose deprivation and the Warburg effect [114]. Yet, the compensatory mechanisms of glycogenolysis and gluconeogenesis that maintain serum glucose levels in the ketogenic diet also apply in fasting [111]. Furthermore, evidence in the present interdisciplinary review suggests that reduction of Pi concentrations in the tumor microenvironment through restricted dietary phosphate intake is a mediating factor linking fasting and effective cancer treatments. Moreover, although fasting is a short-term strategy that may contribute to the restoration of nutritional imbalances, fasting, like a ketogenic diet, is not a substitute for a long-term strategy to prevent cancer with a properly balanced diet that proscribes excessive intake of dietary phosphate.

### 7.2. Tumor Dynamics Model

In 2004, Kuang, Nagy, and Elser used a tumor dynamics model to predict that reducing the phosphorus concentrations of tumor cells by half could lead to a 75% reduction in tumor size [119]. Despite the negative effects associated with ketogenic diets, tumor remission following administration of Hagihara et al.’s low-phosphate ketogenic formula to patients with advanced cancer inadvertently confirms Kuang, Nagy, and Elser’s prediction of tumor reduction through dietary phosphorus restriction. Effects of fasting and fasting-mimicking diets on cancer also indirectly support the benefits of reduced dietary phosphate intake. Furthermore, the U.S. National Cancer Institute described the possibility of conducting a randomized trial based on emerging evidence of reduced cancer risk associated with a dietary component [120], and future trials should investigate dietary phosphate restriction as a treatment for tumor remission in patients with cancer.

**Figure 1:**
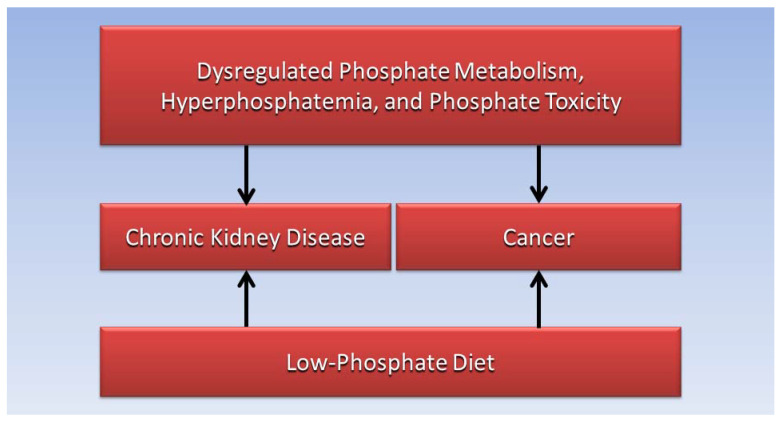
A low-phosphate diet for CKD should be tested to treat cancer, based on common factors of dysregulated phosphate metabolism, hyperphosphatemia, and phosphate toxicity.

The answer to the question posed in the title of this paper, “can a low-phosphate diet for chronic kidney disease treat cancer?”, lies in breakthrough knowledge synthesized from extant evidence in the present interdisciplinary literature review combined with new evidence from findings in future clinical research. Figure 1 shows that dysregulated phosphate metabolism, hyperphosphatemia, and phosphate toxicity are common factors in CKD and cancer, and the figure shows that a low-phosphate diet for CKD should be tested to treat cancer.

## 8. Conclusions

Interdisciplinary research is needed to develop new strategies to prevent and treat cancer. Dysregulated phosphate metabolism and phosphate toxicity are associated with tumorigenesis, and world-wide agency recommendations to reduce cancer risk factors are consistent with effects of phosphate toxicity in cancer. A low-phosphate diet is used by patients with CKD to manage high serum-phosphate levels, and, in addition to fasting, a ketogenic diet that is low in phosphorus is effective in tumor remission in patients with advanced cancer. However, ketogenic diets have adverse effects including raised risks of cardiovascular disease. Many cancer patients seek dietary advice, but to the best of our knowledge, an anti-cancer response to a diet low in phosphate has not been clinically tested in humans. Future research should investigate the role of a low-phosphate diet in reducing tumors and preventing tumor recurrence in patients with cancer.

## Figures and Tables

**Table 1:** WCRF/AICR recommendations for cancer prevention and additional associations with phosphate toxicity.

WCRF/AICR Recommendations for Cancer Prevention	Additional Associations with Phosphate Toxicity
“Be a healthy weight—Keep your weight within the healthy range and avoid weight gain in adult life”	Obesity is linked to higher dietary phosphate intake and increased risk of certain cancers [30].
“Be physically active—Be physically active as part of everyday life—walk more and sit less”	During exercise, phosphorus shifts from intracellular areas to extracellular fluid [60] for removal by the kidneys, and phosphate removal from blood during hemodialysis is increased with aerobic exercise [61].
“Eat a diet rich in wholegrains, vegetables, fruit and beans—Make wholegrains, vegetables, fruit and pulses (legumes) such as beans and lentils a major part of your usual daily diet”	Plant-based diets are generally lower in phosphorus compared to meat-based and dairy-based diets [62].
“Limit consumption of ‘fast foods’ and other processed foods high in fat, starches or sugars—Limiting these foods helps control calorie intake and maintain a healthy weight”	Ultra-processed food consumption with highly absorbable phosphate food additives is increasing [63].
“Limit consumption of red and processed meat—Eat no more than moderate amounts of red meat, such as beef, pork and lamb. Eat little, if any, processed meat”	Meat is naturally high in phosphorus [62].
“Limit consumption of sugar sweetened drinks—Drink mostly water and unsweetened drinks”	Phosphoric acid is a common additive in sugar-sweetened beverages like colas [63].
“Limit alcohol consumption—For cancer prevention, it’s best not to drink alcohol”	Non-traumatic rhabdomyolysis from alcohol intake increases serum Pi [35].
“Do not use supplements for cancer prevention—Aim to meet nutritional needs through diet alone”	Phosphorus is added to some dietary supplements [64]. Vitamin D supplements also do not lower cancer risk associated with high dietary phosphate [65].
“For mothers: breastfeed your baby, if you can—Breastfeeding is good for both mother and baby”	Breast milk is six-times lower in phosphorus compared to cow’s milk [66], and increased need for phosphate during pregnancy and lactation may mitigate effects of excessive dietary phosphate intake [35].

## Data Availability

No new data were created or analyzed in this study. Data sharing is not applicable to this article.
